# Resisting the gallium “Trojan horse”: Experimental evolutionary insights into microbial resistance

**DOI:** 10.36922/gpd025290054

**Published:** 2025-11-28

**Authors:** Akamu Jude Ewunkem

**Affiliations:** Department of Biological Sciences, School of College of Arts, Sciences, and Education, Winston-Salem State University, Winston-Salem, North Carolina, United States of America

**Keywords:** Gallium, Greek gift, Trojan horse, Iron, Pathogens, Experimental evolution

## Abstract

Given the increasing challenge of multidrug-resistant infections, there is a significant push to explore alternative treatments beyond conventional antibiotics. Historically, heavy metals have served as antimicrobial agents, and the current review focuses on elucidating their mechanisms of action and effectiveness against multidrug-resistant pathogens. Among these, gallium has emerged as a particularly promising antimicrobial agent with the potential to address the growing threat of antibiotic resistance. Using a “Trojan horse” strategy, gallium leverages its chemical similarity to iron to deceive pathogens. Because their ionic properties are nearly identical, pathogens readily import gallium through their native iron uptake systems. Once inside, the gallium is unable to perform iron’s necessary functions and instead acts as a poison, disrupting the microbe’s metabolism and ultimately causing its death. However, the question remains whether pathogens might adapt genetically, potentially leading to more harmful strains resistant to gallium’s effects. Given the significant threat posed by multidrug-resistant pathogens, such as *Escherichia coli*, *Staphylococcus aureus*, and *Candida tropicalis*, this review explores new approaches to combating antimicrobial resistance. This hybrid review uses experimental evolution to identify the adaptive mechanisms that allow bacteria and *Candida* to overcome gallium’s therapeutic “Trojan horse” effect. Studying gallium resistance is important for medical applications, as it provides critical insights into combating drug-resistant pathogens.

## 1. Introduction

While the feeling of receiving a gift is generally positive, it is not universally “loved.” People might decline a gift for various reasons, often stemming from personal beliefs, past experiences, or anxieties about the implications of receiving something. To acknowledge his advisor’s significant guidance during his graduate program, a student gave a special gift. However, the advisor returned the gift within 24 h, citing concerns for his personal safety or well-being. This fear might stem from a perceived threat, a previous bad experience, or a feeling of danger associated with the gift or the person who gave it. This describes the source of a fear or anxiety related to receiving gifts, drawing a parallel to the legend of the “Trojan horse” or “Greek gift,” by which the Greeks shrewdly vanquished the Trojans, leading to the fall of Troy.

The story of the fall of Troy, as recounted by the Greek poet Homer, involved the advice of the Goddess Athena to Epeius, son of Panopeus. He was instructed to build a gigantic wooden horse, in the belly of which the bravest Greek warriors concealed themselves under the direction of Odysseus.^1^ The Greeks burned their camp, sailed away, and hid their ships behind a nearby island. The Trojans emerged from behind their walls to see a massive wooden horse, and in their joy at the Greeks quitting the war, they foolishly brought the horse (which they saw as a testament to their greatness) inside the walls of Troy. After that, the Greek soldiers hidden inside emerged, opening the city gates to the rest of the Greek army, and sacked Troy.

The notion of the “Greek gift” or the “Trojan horse” serves as a metaphor for gifts that may conceal ulterior motives or harmful consequences, and has become throughout human history as the symbol of something that is too good to be true. For example, in chess, the classic bishop sacrifice, in which the white player exchanges their bishop for the pawn on black’s H7 square, is also called the “Greek gift.” The same principle is applied in biological and chemical strategies, such as embedding a poisonous substance within an attractive carrier, as seen in ant baits. The ants consume the bait, which contains a slow-acting poison, and carry it back to their nest, where they share it with other ants. Similarly, gallium quenching employs this approach to curb the proliferation of harmful microbes.

**Figure 1.**
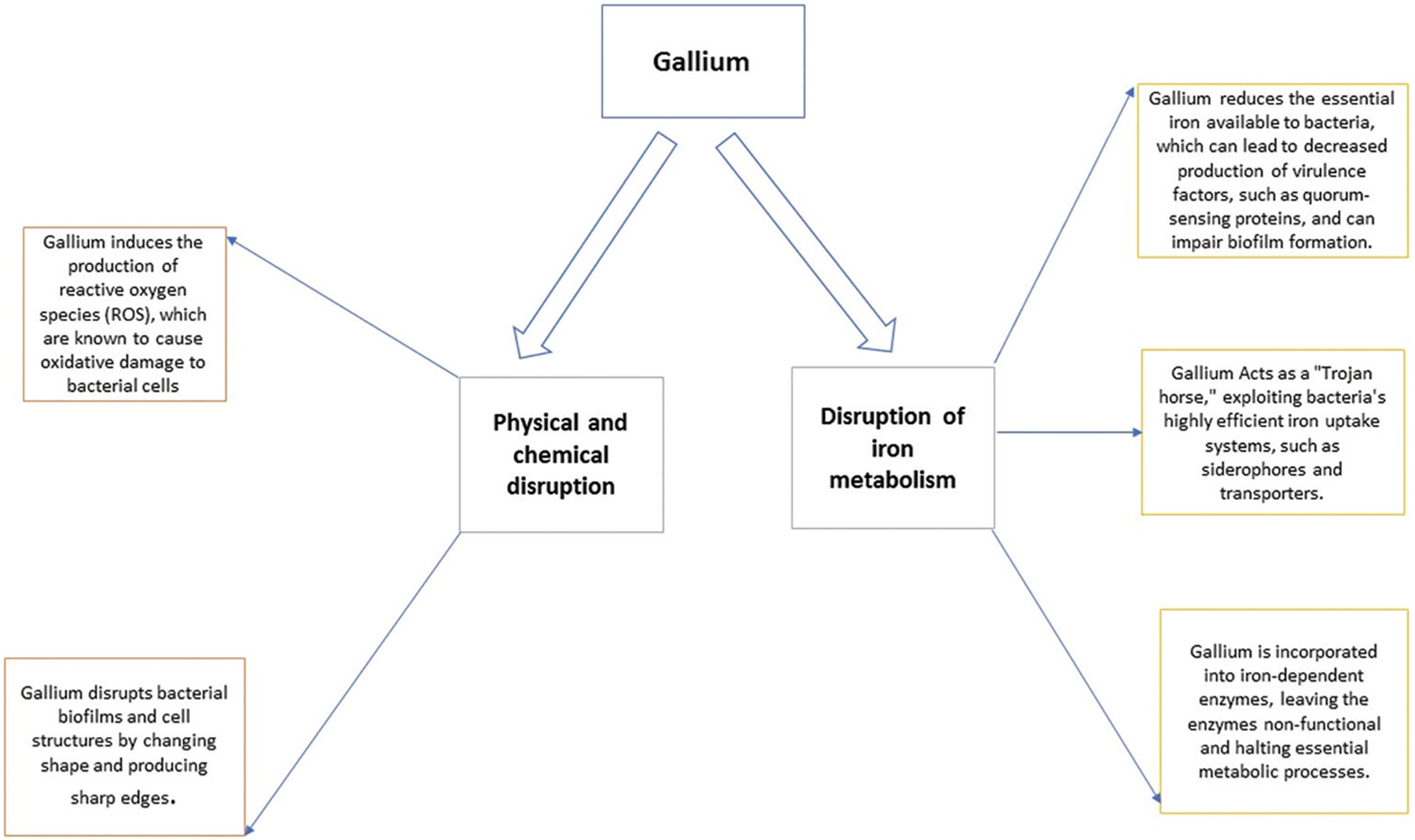
Antimicrobial activity of gallium. Image created by the author.

Gallium is emerging as a promising metal-based antimicrobial agent, particularly effective against multidrug-resistant pathogens.^2,3^ The mechanism of action of gallium involves mimicking iron and disrupting bacterial iron metabolism, leading to inhibited growth and potentially preventing the development of drug resistance.^4^ In addition, gallium can interfere with iron-dependent enzymes by replacing iron in them, effectively disrupting bacterial metabolism (Figure 1).^5^ Interest in gallium-based therapies reflects the broader search for novel approaches to control the spread of multidrug-resistant pathogens. Traditional methods utilize antibiotics and antimicrobial agents that kill large proportions of bacterial populations. Unfortunately, this method stimulates the evolution of drug-resistant strains as individuals with mutations that confer resistance to a particular antibiotic substance rapidly overtake the population.^6,7^

Gallium is known to exhibit antimicrobial properties, effectively inhibiting the growth of bacteria, such as *Streptomyces pilosus*, *Pseudomonas aeruginosa*, *Klebsiella pneumoniae*, *Escherichia coli*, and *Staphylococcus aureus*.^8,9^ The potential of gallium-based compounds as next-generation antimicrobials highlights the importance of understanding and addressing the evolution of resistance to gallium. This review integrates findings from both literature and experimental evolution studies to explore whether pathogens, including bacteria and fungi, can develop resistance to gallium, an iron mimic. Essentially, experimental evolution offers a potent means to monitor, define, and foresee how pathogens develop resistance to innovative antimicrobials like gallium, ultimately leading to improved methods for tackling drug-resistant infections. This review incorporates the author’s experimental observations to examine how *E. coli*, *S. aureus*, and *Candida tropicalis* adapt to gallium and iron, with particular attention to genomics and morphological changes. An evolutionary study on pathogen resistance should consider genomics and morphological changes as central factors.^10^ These research findings underscore the significant impact of gallium and other metal-based antimicrobial substances on the environment. Understanding how pathogens, such as *E. coli*, *S. aureus*, and *C. tropicalis*, develop resistance to gallium offers crucial insights into the broader mechanisms of antimicrobial resistance.

## 2. Experimental evolution

**Figure 2.**
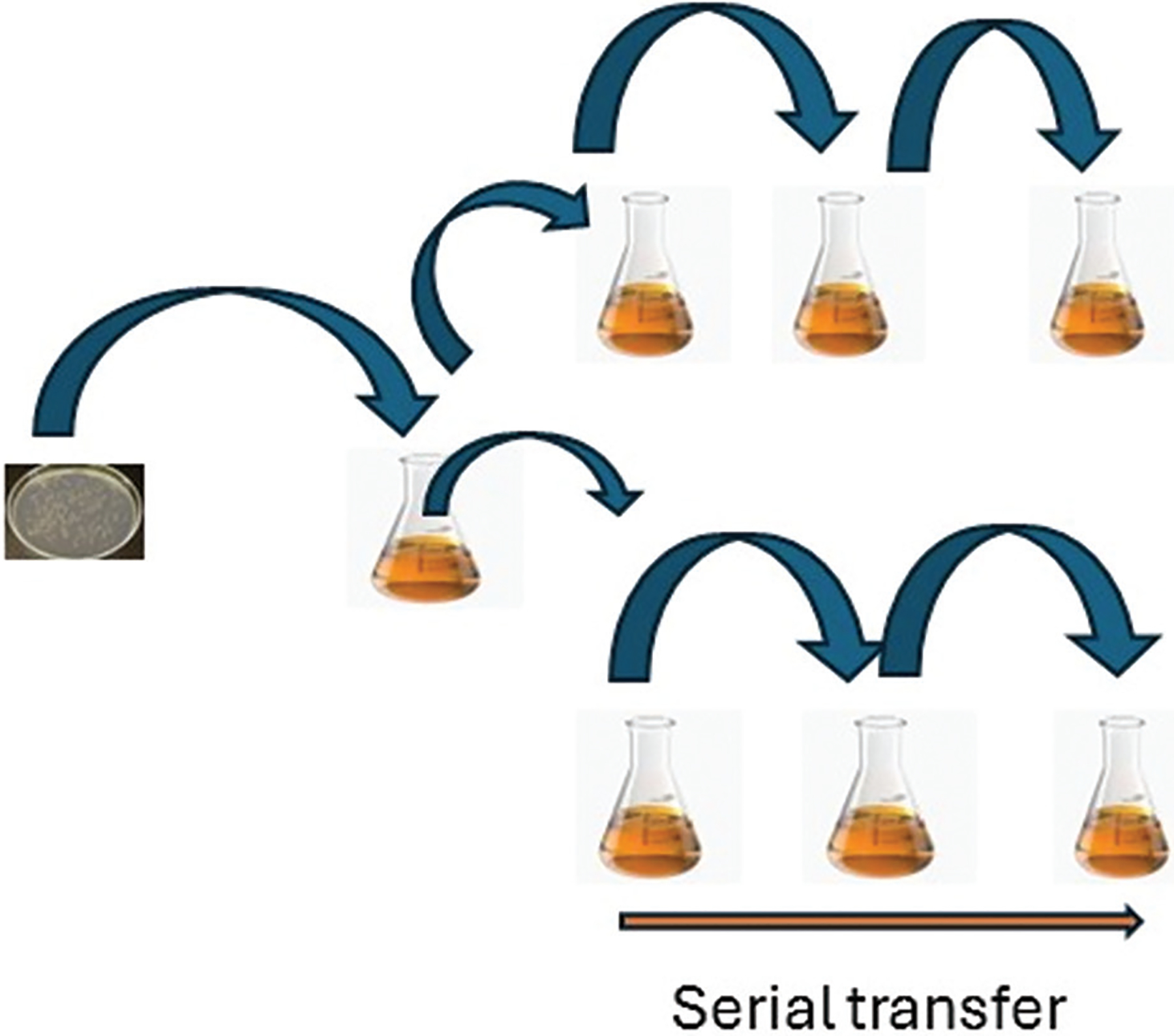
Schematic diagram of the serial dilution and transfer process used for experimental evolution. Image created by the author.

Experimental evolution is a laboratory method used to study how populations, such as bacteria and other pathogens, adapt to certain conditions over time. In studies of antibiotic resistance, a common “serial transfer” protocol enables researchers to monitor the emergence of resistance over successive generations (Figure 2).^11–15^ Continuous exposure of bacterial populations to antimicrobial agents allows researchers to track the genetic mutations and phenotypic adaptations underlying resistance. A well-known example is Richard Lenski’s long-term evolution experiment, which has tracked genetic changes in *E. coli* for over 80,000 generations. This study and other similar studies provide valuable insights into the evolutionary dynamics of drug resistance.

The methodology of experimental evolution has distinct strengths but also inherent limitations. Experimental evolution is highly advantageous for studying evolutionary processes in real time, particularly with microbes that have rapid generation times.^16^ This approach provides a controlled laboratory environment, enabling researchers to precisely monitor and analyze adaptation, genetic changes, and organismal reactions to selective pressures. A notable application is in the study of drug resistance, where it allows for long-term observation of how mutations lead to increased resistance and potential fitness trade-offs.^17,18^ By generating crucial data on adaptation in a simplified setting, experimental evolution offers insights that would be challenging to obtain from complex, natural ecosystems.^19–21^ This capability is instrumental for understanding resistance development and strategizing to mitigate its emergence.

Despite the advantages of experimental evolution, it also has certain disadvantages. Experimental evolution is constrained by its simplified nature, which limits time scales and population sizes. In addition, laboratory environments may not accurately reflect complex natural conditions.^11^ This oversimplification can lead to misinterpretation of selective forces and other processes at work, as laboratory adaptations may come with trade-offs that cause disadvantages in more complex or different environments. The methodology also has limitations in detecting mutations, particularly in population-level sequencing, and can struggle to isolate specific mutations from others that may have accumulated over time.^19^ Finally, experimental findings can be limited by the historical genetic variation present at the start of the experiment, which can prevent evolution from proceeding in all potential directions.^22^ This review examines the evolution of pathogen resistance to gallium, focusing on insights gained from experimental evolution.

## 3. Experimental evolution of gallium resistance

Gallium (Ga, an atomic number 31) is a chemical element discovered by the French chemist Paul-Émile Lecoq de Boisbaudran in 1875.^23,24^ Since then, its applications have been explored across various domains. One significant area of research focuses on gallium’s antimicrobial properties, particularly its ability to interfere with bacterial iron acquisition and metabolism, offering a potential strategy to combat infections, including those caused by drug-resistant strains.^25,26^ Gallium exploits bacteria’s need for iron by entering cells via iron-uptake pathways and disrupting essential iron-dependent processes, thus preventing bacterial growth.^27^ In essence, gallium functions as a biological “Trojan horse” or “Greek gift”: it is initially accepted as something beneficial, but it ultimately leads to destruction from within.^28,29^ Gallium deceives bacteria into taking it up, mistaking it for iron, which is essential for their growth and survival.

Given the increasing concern over antibiotic resistance, understanding how microbes develop resistance to gallium is essential, as gallium is being explored as a potential new treatment for bacterial infections. Studies using experimental evolution have demonstrated that bacteria can develop resistance to gallium.^29–31^ The development of gallium resistance in bacteria is frequently driven by genetic mutations that affect iron transport, since gallium interferes with bacterial iron metabolism. As a result, these gallium-resistant bacteria may also be resistant to other metals, such as silver and iron, but this does not correlate with resistance to standard antibiotics.^29,30^ The review explores the ways in which *E. coli*, *S. aureus*, and *C. tropicalis* develop resistance to gallium, an antimicrobial that disrupts iron metabolism.

### 3.1. The mechanisms of gallium resistance in *E. coli*

*E. coli* is a rod-shaped, Gram-negative bacterium. It is typically found in the intestines of humans and animals. While many *E. coli* strains are harmless and even beneficial, several strains can cause illness through toxin production or other pathogenic mechanisms. Intestinal infections, such as Diarrhea and dysentery, as well as extraintestinal infections, such as urinary tract infections, pneumonia, meningitis, and bloodstream infections (bacteremia), can all result from these pathogenic strains.^31,32^ Recognizing and promptly treating *E. coli* infections appropriately is vital due to their significant impact on patients and the healthcare system. Antibiotics, such as fluoroquinolones, trimethoprim/sulfamethoxazole, and nitrofurantoin, are often effective against these infections, leading to improved patient outcomes.^33^ However, the increasing prevalence of antibiotic resistance in *E. coli* presents a significant obstacle to effective treatment. *E. coli* achieves resistance by developing mechanisms to overcome the effects of antibiotics, often by acquiring resistance genes.^34^ Overuse and misuse of these medications, unfortunately, accelerate this evolutionary process.^35^ In laboratory and animal model research, gallium has shown promise as an antibacterial agent against *E. coli* by interfering with its iron metabolism.^36^ Gallium’s antibacterial effects stem from its ability to mimic iron, leading to its uptake and incorporation into crucial iron-dependent enzymes and proteins in *E. coli*, thereby impairing bacterial function and impeding growth.^27^

### 3.2. Experimental evolution protocol and results

Experimental evolution was conducted using *E. coli* K12 MG1655. Briefly, *E. coli* K12 MG1655 colonies were serially diluted and plated from a bacterial suspension onto agar plates to isolate individual colonies. Twelve individual colonies were cultured separately in 10 mL sterile Davis minimal broth (DMB) (Difco, Sparks, US) supplemented with 10% dextrose and 0.1% thiamine hydrochloride in 50 mL conical flasks. Six *E. coli* cultures were designated as the control group, receiving only sterile liquid media, while another six cultures were designated as the treatment group and exposed to gallium over time. A serial transfer protocol was used, in which 0.1 mL of the previous culture was transferred into 9.9 mL of sterile DMB every 24 h. The treatment group received an increasing amount of gallium (20–100 mg/L) over the course of the study. All flasks were incubated in a shaking incubator (Thermo Scientific^™^ Solaris 4000, Fisher Scientific, US) at 37°C, 117 rpm. After 2 weeks, the bacterial cultures were placed on covered slips after 10 min of incubation at room temperature, and the samples were gently removed. Thereafter, the bacteria were fixed with Karnovsky fixative and incubated at 4°C overnight. The samples were dehydrated in ethanol, air-dried, and subsequently sputter-coated to prevent clumping during microscopy. Microscopy was performed with a focused ion beam field emission scanning electron microscope (FIB-FESEM) (Auriga-BU, Carl Zeiss, Germany). The images were acquired at a working distance of 7 mm and an accelerating voltage of 3 kV.

**Figure 3.**
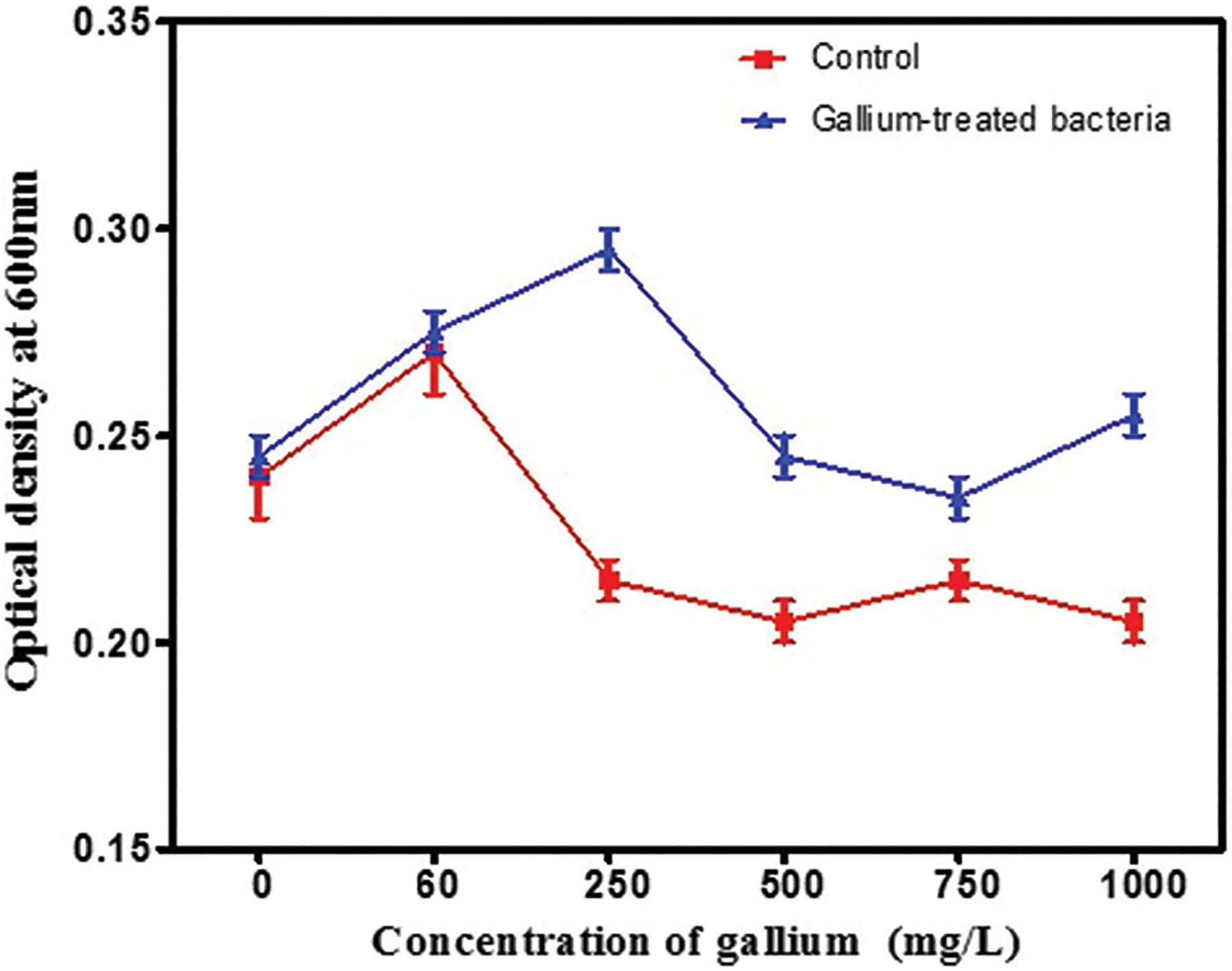
The mean and standard error of the 24-h growth of *Escherichia coli* in increasing concentrations of gallium. The growth of *E. coli* treated with gallium was significantly greater than the controls (*F* = 86.2, *p*<0.0001). Image created by the author.

*De novo* gallium resistance in *E. coli* developed after 10 days of excess gallium treatment, providing evidence for the potential development of gallium resistance (Figure 3).

In another experiment, Graves *et al*.^30^ also demonstrated that prolonged exposure to gallium resulted in *E. coli* developing resistance. Specifically, when *E. coli* is cultured under low gallium concentrations, its resistance increases noticeably within 10 days.^30^ Gallium resistance in bacteria, specifically in *E. coli*, develops through a combination of genetic mutations and physical changes. These changes are often observed in genes related to iron transport and other cellular processes (Table 1). For example, mutations were detected in the iron-citrate transporter gene (*fecA*) and the IS30 transposase gene (*insl1*), as well as intergenic mutations affecting arsenate reductase (*arsC*) and a pseudogene (*yedN*). The majority of these genes are critical for the proper functioning of iron metabolism. These alterations enabled the bacteria to counteract gallium’s effects. The control group also exhibited significant mutations, including an intergenic mutation between *arsC*, *yhiS*, and *rpoB* genes, as well as a gene associated with rifampicin resistance, among others (Table 2).

**Figure 4.**
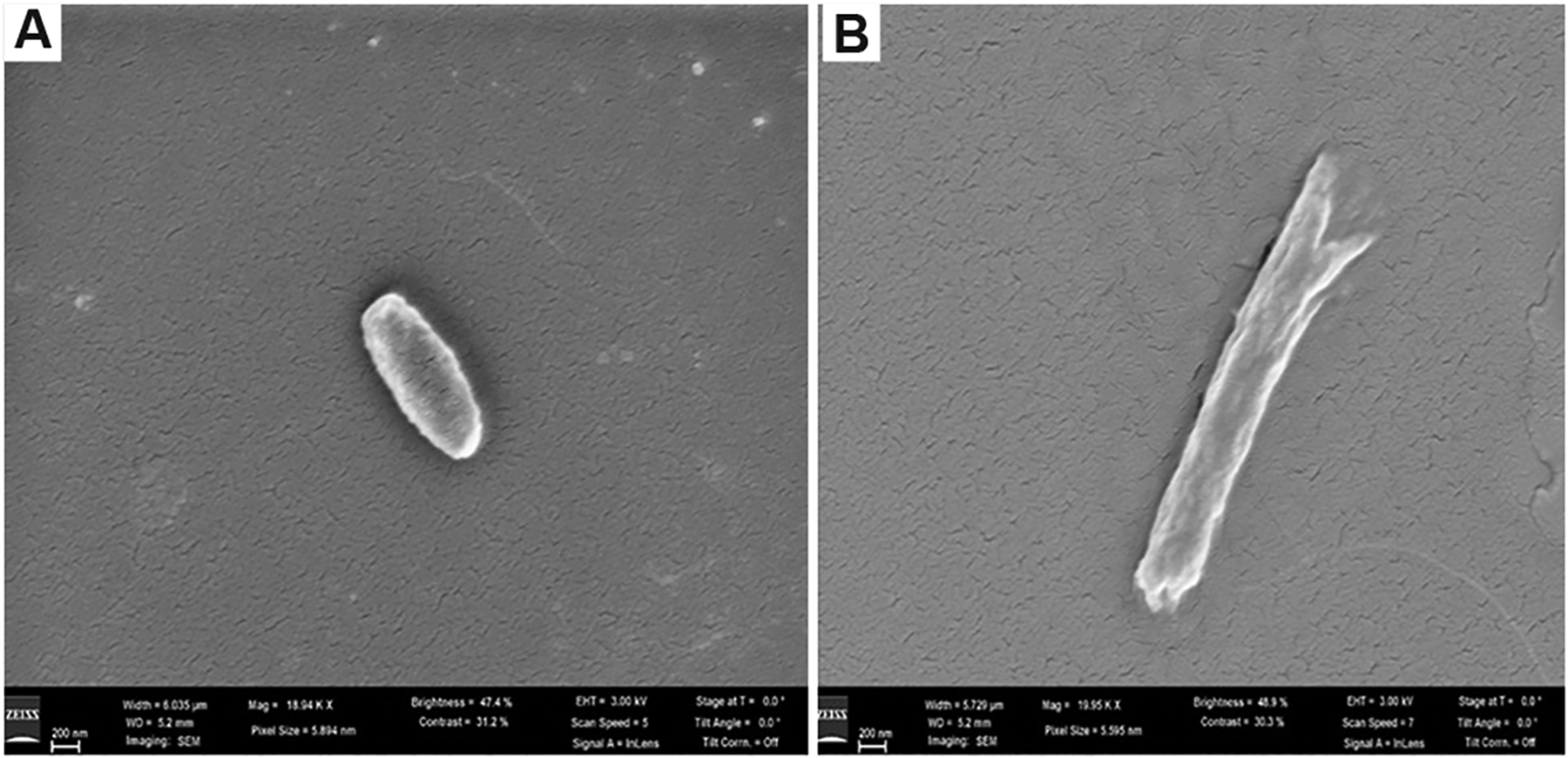
Scanning electron microscopic images. A morphologically typical *Escherichia coli* cell from the control group (left) and an elongated cell with detached cellular membrane (right), observed in greater quantities in the experimental group due to induced stress. Scale bar: 200 nm; magnification: Left: ×18,940; Right: ×19,950. Image obtained by the author.

Scanning electron microscopy (SEM) revealed morphological changes in *E. coli*, confirming an adaptive response to gallium exposure. Gallium exposure in the experimental group induced stress in *E. coli* cells, leading to notable morphological changes, such as elongation and membrane detachment, in contrast to the typical morphology observed in the control group (Figure 4). Bacteria can adapt to heavy-metal stress by undergoing morphological changes, specifically elongation.^37^ To compare gallium resistance mechanisms across major bacterial types, the studies on *S. aureus*, a Gram-positive bacterium, are discussed in Section 3.2.

### 3.3. The mechanisms of gallium resistance in *S. aureus*

*S. aureus*, a Gram-positive bacterium, is a common cause of various human infections, ranging from minor skin problems to severe, potentially fatal illnesses.^38^ The pathogenicity of *S. aureus* stems from its wide range of virulence factors, which enable colonization, immune evasion, and tissue damage within the host.^39^ Common antibiotics used to treat *S. aureus* infections include cefazolin, nafcillin, oxacillin, vancomycin, daptomycin, and linezolid.^40^ Antibiotic resistance in *S. aureus* is a serious problem, as these bacteria can develop resistance through various means, including genetic mutations and the acquisition of resistance genes from other bacteria.^41,42^ Consequently, certain *S. aureus* strains are becoming increasingly difficult to treat with conventional antibiotics.

With the growing concern over antibiotic resistance in *S. aureus*, particularly multidrug-resistant strains, researchers are exploring new treatment options. One promising approach involves the use of gallium as an alternative to traditional antibiotics. Gallium demonstrates antimicrobial activity by disrupting bacterial iron uptake and utilization.^43^ Bacteria require iron for vital processes, such as growth and replication, and can mistakenly uptake gallium due to its chemical resemblance to iron. However, since gallium cannot perform the same biological roles as iron, it essentially starves the bacteria, hindering their essential functions.

**Figure 5.**
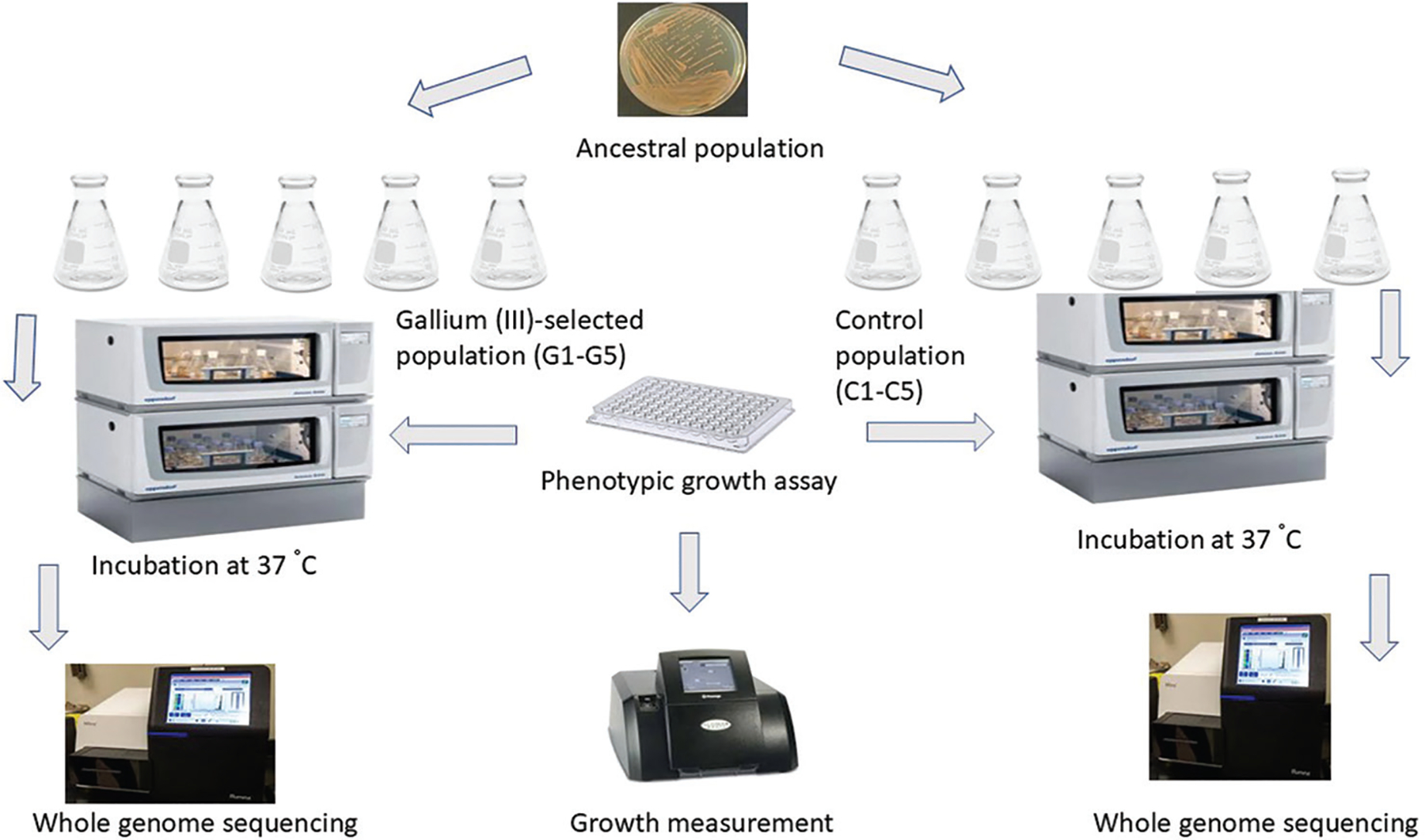
Experimental evolution layout. Reprinted from reference.^29^

Experimental evolution was conducted to induce gallium resistance by culturing bacteria under progressively increasing gallium concentrations. Briefly, the experimental procedure involved a daily passage of the culture for 7 days. A 0.1 mL aliquot of the previous culture was transferred into 9.9 mL of fresh nutrient broth. This step ensured propagation of the cultures before exposure to gallium(III) nitrate for selective pressure. A serial transfer protocol was used to investigate the effect of gallium on *S. aureus*. Ten cultures were divided into a control group (C1-C5) and a treatment group (G1-G5). Both groups were sub-cultured into fresh media every 24 h, and the treatment group received increasing concentrations of gallium (III) nitrate (300 mg/L) over the course of the study. Flasks containing the inoculum were incubated in a shaking incubator at 37°C, 150 rpm (Figure 5). Morphological characteristics of the gallium-resistant, control, and ancestral bacterial strains were evaluated on day 30 using FIB-FESEM imaging. Resistance to gallium was established in a bacterial culture after 20 days of treatment, and the genomic DNA was subsequently isolated and sequenced by SeqCoast Genomics.

**Figure 6.**
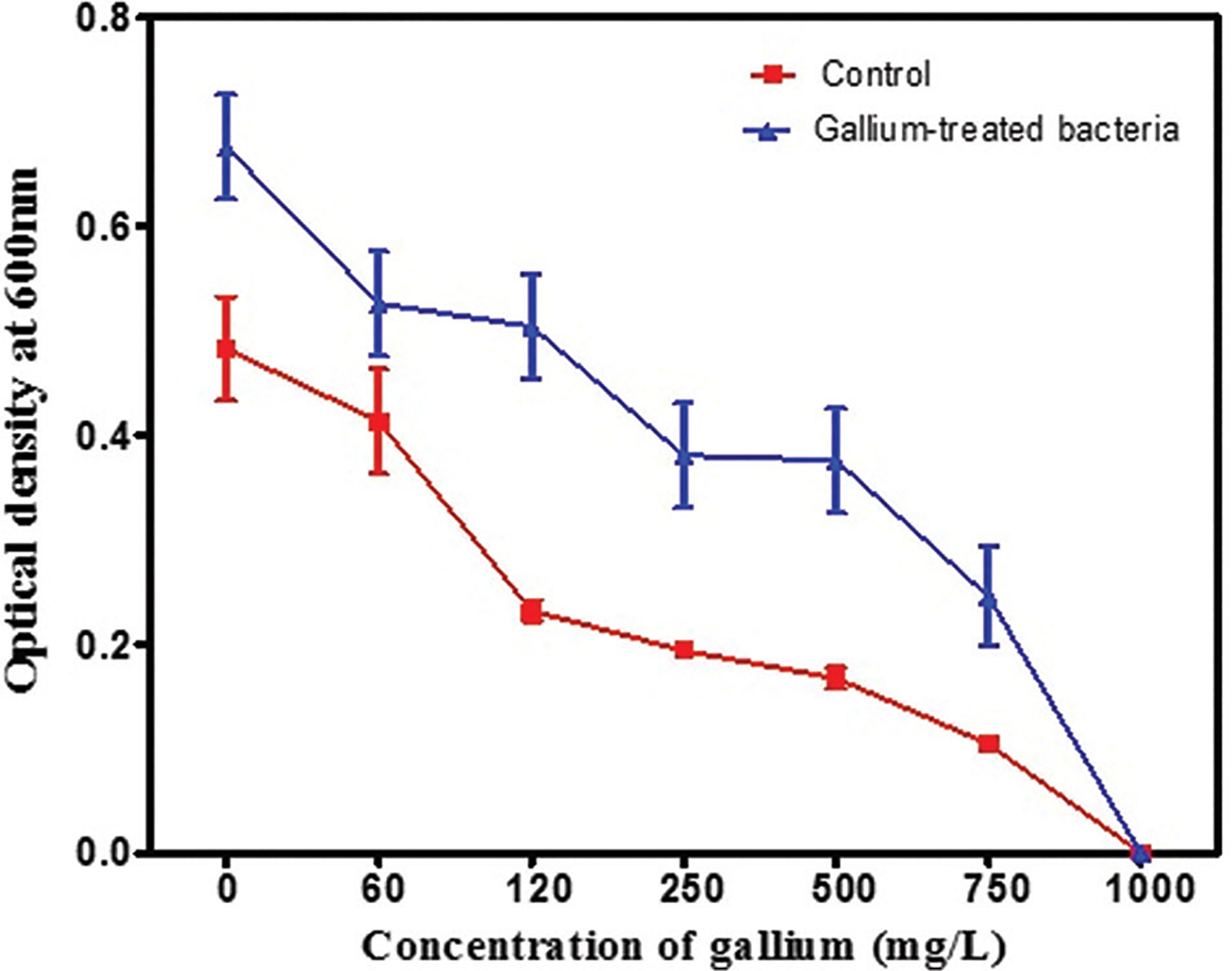
The mean 24-h growth of *Staphylococcus aureus* cultures at increasing concentrations of gallium after 20 days of treatment. Gallium-treated *S. aureus* exposed to 300 mg/L gallium in nutrient agar broth for 20 days exhibited significantly greater growth than the control grown in nutrient broth without gallium.^29^ Reprinted and modified from Reference.^29^

*S. aureus* can develop resistance to gallium if it is used excessively in treatment.^29^ Experimental evolution studies revealed that *S. aureus* can develop resistance to gallium after 20 days of treatment (Figure 6).^29^ This resistance is acquired through mutations and morphological changes.

**Figure 7.**
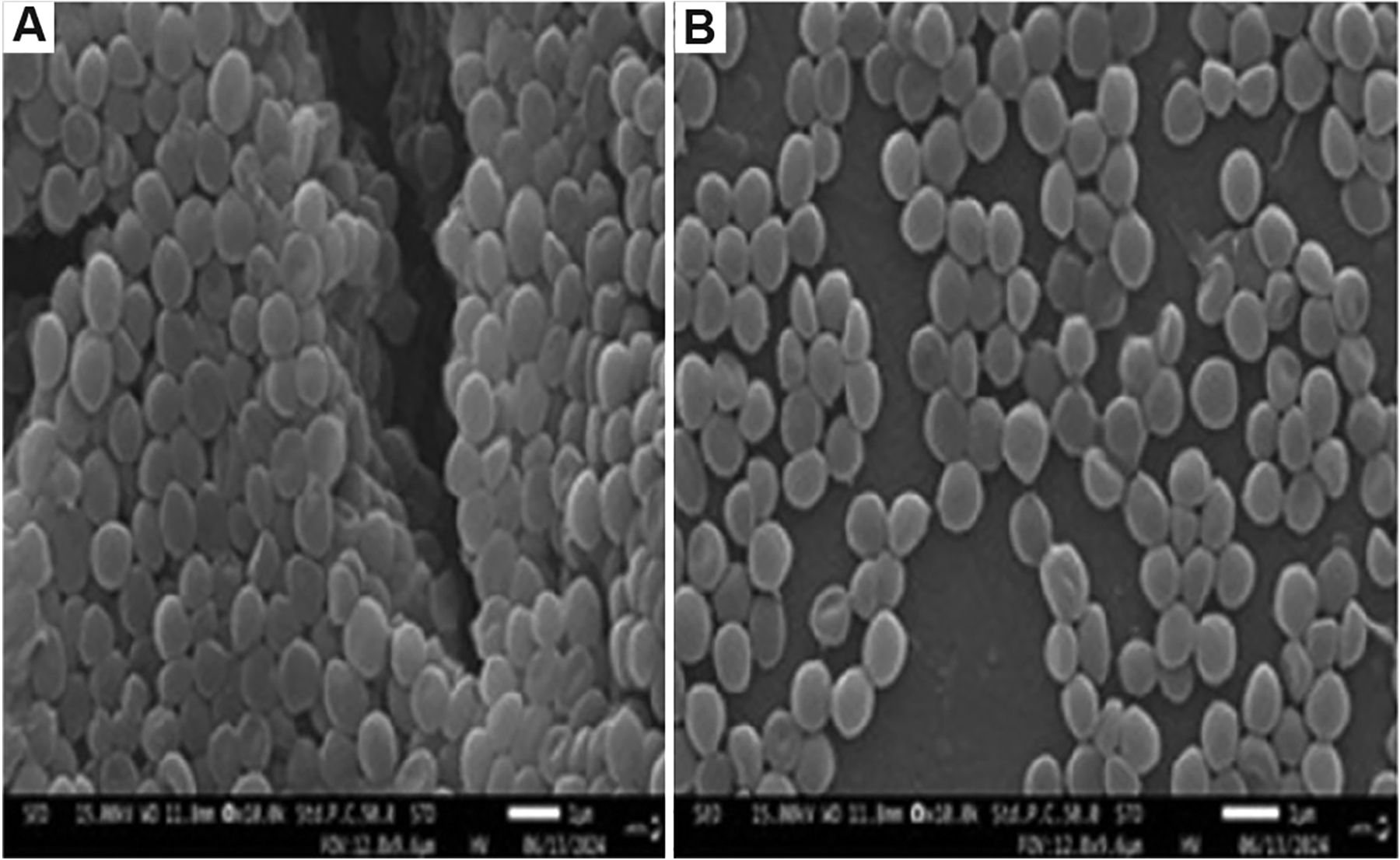
Scanning electron micrographs of *Staphylococcus aureus* cultures under gallium treatment. (A) Intact, smooth, and spherical appearance of control bacterial cultures. (B) The significant morphological alterations in the bacterial cultures after 20 days of treatment with gallium (III) nitrate, resulting in various cell shapes, including oval, triangular, rod-like, club-shaped rods, and vibrio-shaped.^29^ Scale bar: 1 μm; magnification: 15.00 KV; ×10,000. Reprinted and modified from Reference.^29^

Whole-genome analyses of all gallium-treated *S. aureus* revealed the presence of polymorphisms within the genes encoding the staphyloferrin A export major facilitator superfamily transporter (*KQ76_RS01520*), teichoic acid D-alanine esterase (*fmtA*), adenine phosphoribosyltransferase (*KQ76_RS08360*), peptidoglycan teichoic acid D-alanyltransferase (*dltB*), magnesium transporter CorA family protein (*graR*), general stress protein (*KQ76_RS01815*), and a hypothetical protein (*KQ76_RS09255*) (Table 3). Mutations in these genes are crucial for *S. aureus* to survive in toxic metal-contaminated environments by maintaining cellular homeostasis and mitigating metal-induced oxidative stress.^29,44–46^ Polymorphisms were identified in several genes within the control, affecting key cellular functions. These genetic variations included: Phosphate transport (*pstC*), acyltransferase family protein (*KQ76_RS04365*), pP2C phosphatase (*KQ76_RS10525*), 5-oxoprolinase/transcription elongation factor (*pxpB/greA*), *ica* operon regulator (*icaR*), and cell elongation protein (*cozEb*). These polymorphisms regulate processes, such as cell division, antimicrobial resistance, nutrient acquisition, and the expulsion of harmful substances.^47–49^ The interaction of gallium(III) nitrate with *S. aureus* cells was studied using SEM. Exposure to gallium(III) nitrate for 20 days resulted in unexpected variation in cell shapes, unlike the consistently spherical control and ancestral cells (Figure 7).^29^ This finding suggests that prolonged stress triggers cellular adaptations, such as changes in size and shape, that enhance bacterial survival under toxic conditions.^50^

According to Ewunkem *et al*.,^29^ even in the absence of gallium exposure, control bacteria exhibited survival strategies similar to the gallium-treated bacteria through spontaneous polymorphism sweeps. The mutations were found in genes that regulate essential cellular processes, including membrane biogenesis, stress responses, cell proliferation, and virulence. Key polymorphisms affected an acyltransferase family protein (*KQ76_RS04365*), a protein phosphatase 2C family serine/threonine phosphatase (*KQ76_RS1052*), and the 5-oxoprolinase subunit PxpB/transcription elongation factor GreA (*pxpB*/*greA*) (Table 4). This indicates that genetic variations, which play a significant role in adapting to environmental changes, were already present within the bacterial population.

Variations in bacterial genes can significantly affect bacterial shape by altering proteins involved in cell wall structure and division.^51^ According to a recent study from Ewunkem *et al*.,^29^ gallium exposure for 20 days resulted in morphological changes in *S. aureus*. These findings suggest gallium’s potential in changing bacterial morphology, likely by affecting cell wall synthesis or division.

Stress-induced morphogenesis is an adaptive process where bacteria alter their cell surface properties, potentially through mechanisms such as genetic polymorphism in *dltB*, to survive and thrive in challenging environments.^17^ The *dltB* gene, found in Gram-positive bacterial cell walls, encodes for teichoic acid D-alanyl transferase, and mutations in this gene are associated with changes in cell surface characteristics.^52^ This adaptation can improve bacterial fitness, enabling them to evade immune cells, resist antimicrobial agents, and persist in biofilms.^53^ Gallium, by mimicking iron, can interfere with crucial cellular processes within various microorganisms. While previous discussions have focused on bacterial mechanisms of gallium resistance, the following section examines how the fungus *Candida* develops resistance to gallium.

### 3.4. The mechanisms of gallium resistance in *C. tropicalis*

As one of the most virulent *Candida* species, *C. tropicalis* has the propensity to invade submucosal blood vessels, leading to invasive candidiasis.^54^ Invasive candidiasis is typically treated with first-line antifungal therapies such as amphotericin B or echinocandins, while extended-spectrum triazoles serve as acceptable alternatives.^55^ However, the efficacy of these treatments is challenged by the emergence of drug resistance.^56^ Specifically, instances of resistance to azole drugs have been documented in *C. tropicalis*. This growing problem necessitates the exploration of new therapeutic avenues. In this context, gallium has shown promise by exhibiting antifungal activity against *C. tropicalis in vitro*.^56^ It is important to investigate whether *C. tropicalis* can develop resistance to gallium, particularly through experimental evolution. This understanding is crucial for assessing gallium’s viability as a treatment for *Candida* infections.

A clinical isolate of *C. tropicalis* was cultured in 10 mL of yeast extract-peptone-dextrose broth overnight at 35°C with 150 rpm agitation. Serial dilution was performed, followed by 24 h incubation of the ancestral *C. tropicalis* to isolate single colonies. Ten unique colonies were cultured separately. The strain was propagated daily by transferring 0.1 mL of the overnight culture into 9.9 mL of fresh, sterile nutrient broth. Five cultures were used for gallium treatment. These replicates were exposed to 1,500 mg/L gallium(III) nitrate (designated G1–5). The concentrations utilized to initiate the experiment were determined by the minimum inhibitory concentration assay. The controls (designated C1-C5) were cultured in nutrient broth.

#### 3.4.1. Determination of minimum inhibitory concentration of gallium

Minimum inhibitory concentration (MIC) values for gallium against *C. tropicalis* were determined using broth microdilution in a 96-well microtiter plate format. The growth of *C. tropicalis* was assessed by measuring the optical density at 600 nm. Ten different concentrations of gallium (0–5,000 mg/L) were used in triplicate to treat the cultures, which were incubated at 37°C for 24 h with agitation at 150 rpm. The MIC was determined to be 1,500 mg/L. This concentration was selected for subsequent experiments because it allowed initial microbial growth without killing the cultures.

#### 3.4.2. Phenotypic assays after 24 h growth

To test whether gallium resistance in *C. tropicalis* confers cross-resistance to iron(III), previously evolved gallium-resistant and control yeast cultures were exposed to various iron(III) concentrations. After a 24-h incubation at 35°C, growth was quantified by measuring optical density at 600 nm using a microplate spectrophotometer, with measurements taken at both the start and end of the incubation.

#### 3.4.3. Preparation of cells for scanning electron microscope

SEM is widely used to assess morphological changes in pathogens.^57^ The morphology of gallium-resistant *C. tropicalis* was analyzed using FIB–FESEM and compared to a control group. Samples were prepared by fixing, dehydrating, and sputter-coating them before imaging. The images were captured using FIB-FESEM at 3 kV with a 7 mm working distance.

#### 3.4.4. Whole-genome sequencing of drug-resistant C. tropicalis isolates

The genomic DNA from each culture was sequenced after 30 days of treatment. Samples were processed by SeqCoast Genomics in accordance with Ewunkem *et al*.’s^29^ protocol, which involved extracting DNA with a DNeasy 96 PowerSoil Pro kit (47017, Qiagen, USA) and preparing sequencing libraries with the Illumina DNA Prep Tagmentation kit (cat# 20015828, Illumina, USA). A sequencer (NextSeq 2000, Illumina, United States) was then used to sequence the libraries. The DRAGEN software (v4.2.7, Illumina, United States) was used to analyze the raw data.

### 3.5. Results and discussion

**Figure 8.**
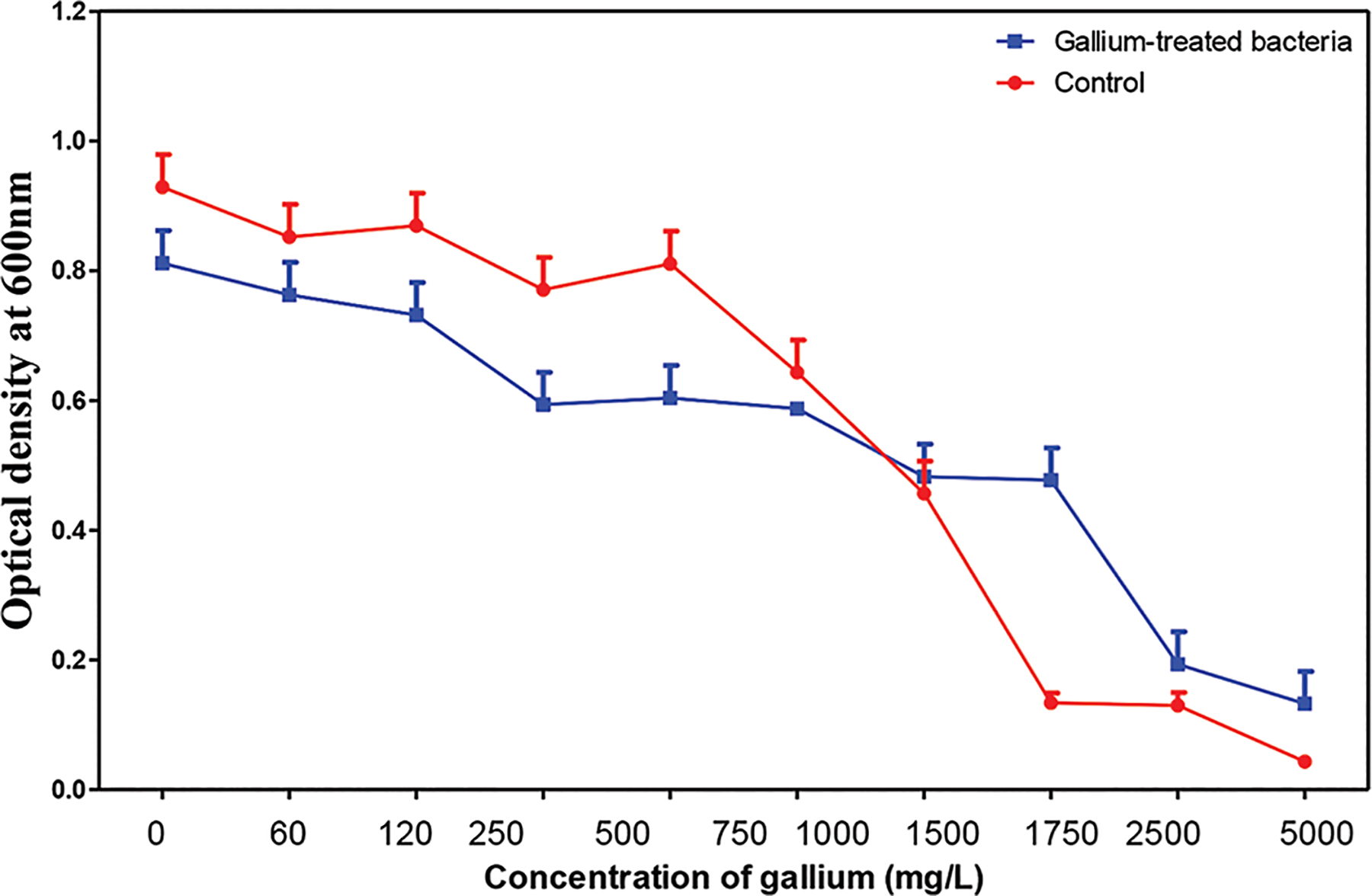
The mean 24-h growth of *Candida tropicalis* cultures in increasing concentrations of gallium after 30 days of treatment. Gallium-treated *C. tropicalis* cultures exposed to 5,000 mg/L gallium in nutrient agar broth for 30 days exhibited significant growth compared to controls grown in nutrient broth without gallium. Image created by the author.

**Figure 9.**
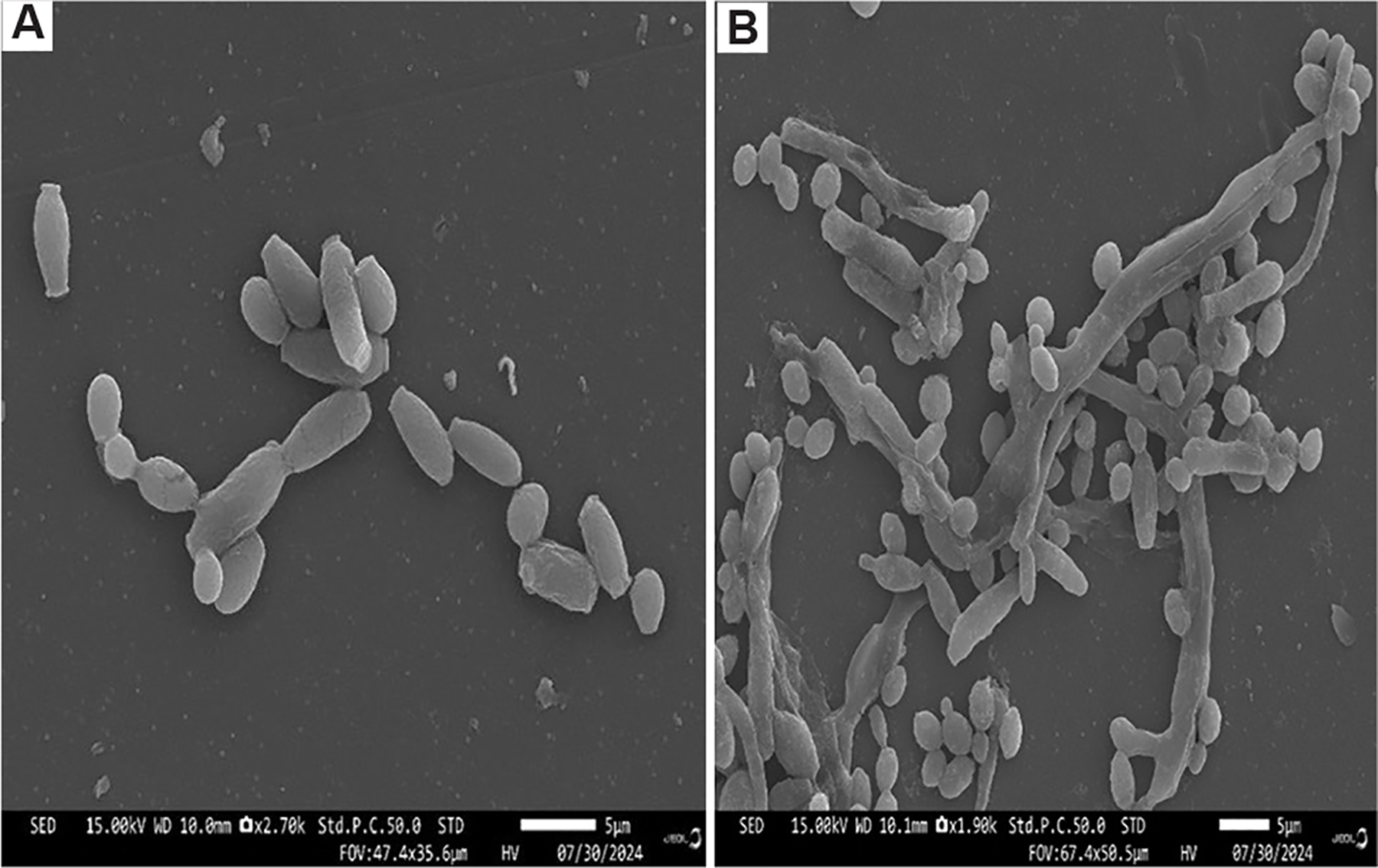
Scanning electron micrographs of *Candida tropicalis* cultures treated with gallium. (A) Untreated control cells with a typical spherical-to-ovoid morphology. (B) After 30 days of gallium(III) nitrate exposure, *C. tropicalis* exhibits significant morphological changes, adopting an elongated, filamentous hyphal form, indicating the presence of biofilms (composed of yeast, hyphal, and pseudohyphal elements). Scale bar: 5 μm; magnification: A = 15.00 KV; ×2,700; B = 15.00 KV; ×1,900. Image obtained by the author.

This experimental evolution study found that *C. tropicalis* can acquire resistance to gallium after continuous exposure over a period of more than 30 days. Specifically, the gallium-treated fungal cultures exhibited significantly greater growth at higher gallium concentrations compared to untreated controls (*p*<0.0001) (Figure 8). A prominent morphological response associated with this resistance was the increased presence of filamentous forms. Filamentation, a transition from the yeast form to hyphal or pseudohyphal growth (Figure 9), is a well-established adaptive strategy employed by *Candida* species under environmental stress.^58–61^ This process is regulated by signaling pathways such as the mitogen-activated protein kinase pathway and enhances the organism’s ability to invade host tissues, evade immune defenses, and form biofilms. The enrichment of filamentous forms in gallium-exposed *C. tropicalis* suggests that morphological adaptation contributed to the observed increase in gallium tolerance.

Whole-genome sequencing revealed that both control and treated bacterial samples harbored mutations in similar genes, complicating the interpretation of gallium’s impact on resistance development (Table 5). Although *C. tropicalis* acquired mutations in genes, such as staphyloferrin A export MFS transporter (*sfaA*), D-ornithine-citrate ligase (*sfaD*), adenine phosphoribosyltransferase (KQ*76_RS08360*), and teichoic acid DAla esterase (*fmtA*), which are known to be involved in metal detoxification and resistance, its overall morphology remained similar to that of control samples. This suggests that the genomic changes, while affecting the ability to handle metals, did not significantly alter cell development and structure to cause notable morphological differences. The presence of gallium resistance in *C. tropicalis* after 30 days of experimental evolution warrants further investigation into the mechanisms governing its susceptibility to this metal. Gallium resistance in *C. tropicalis* can arise from non-heritable or temporary changes at the cellular or phenotypic level, rather than from genetic mutations. These non-genomic mechanisms, which alter a cell’s characteristics without changing its DNA, include:^62,63^

- Epigenetic modifications: Exposure to gallium can induce changes to gene expression without altering the DNA. For example, histones, proteins that package DNA in *Candida*, can undergo modifications such as acetylation or methylation, which alter gene expression and thereby the response to metal stress.^64^
- Biofilm formation: Biofilms are structured communities of microbial cells enclosed in an extracellular polymeric substance. When *Candida* propagates in a protective colony or biofilm, it is much more resistant to heavy metals than when it is alone.^65^ This protective group-based lifestyle was observed in *C. tropicalis* when it was exposed to gallium (Figure 7). Biofilm formation can lead to distinct patterns of cellular differentiation, such as changes in the yeast-to-hyphal transition, thereby enhancing metal resistance.^66^
- Sequestration in the matrix: The extracellular matrix of the biofilm can adsorb and chelate metal cations, preventing the metals from reaching the cells.
- Oxidative stress responses: Exposure to toxic metals, including heavy metals, induces the formation of reactive oxygen species inside the cell. *Candida* can upregulate oxidative stress-mitigating systems to tolerate these effects.^67^
- Bioreduction: *Candida* can reduce metal ions into their less toxic elemental forms, which can then be sequestered inside the cell.^68^
- Metallothionein-like proteins: Stress responses can increase levels of metallothionein-like proteins, which are capable of binding and sequestering excess intracellular heavy metals.^69^
- Quorum sensing: This is a system of cell-to-cell communication that allows microbial communities to coordinate behavior. Exposure to gallium influences quorum sensing, which in turn regulates dimorphism (yeast-hyphae transition) and biofilm formation, both of which are linked to resistance.^70^
- Efflux pumps: While often associated with genetic mutations, the expression of multidrug efflux pump proteins upon exposure to gallium can be regulated at the gene expression level without a genomic change. Overexpression of these pumps actively removes toxic compounds, including heavy metals, from the cell.^71^

In summary, while gallium’s effectiveness in therapeutic strategies often stems from its iron-mimicking properties, this similarity also opens the door to cross-resistance when the target cells or organisms have adapted to handle or resist gallium. Due to the structural similarities between gallium and iron, and gallium’s reliance on iron uptake pathways, understanding how resistance to iron influences the effectiveness of gallium-based therapies is crucial.

## 4. Experimental evolution as a window into iron resistance

Iron is an essential micronutrient with diverse biological functions. It is a key component of various proteins, such as hemoproteins, iron-sulfur cluster proteins, and diiron and mononuclear enzymes.^72^ These proteins are critical for processes like nitrogen fixation, electron transfer in respiration, and overall metabolism.^73^ Acquiring iron is a major challenge and a common nutritional stress for bacteria, even though the element is essential for their metabolic processes.^74–77^ This is primarily due to iron’s low bioavailability and its inherent toxicity in high concentrations. Microbes must manage this paradox by evolving mechanisms to efficiently scavenge iron while avoiding its toxicity.^78,79^

**Figure 10.**
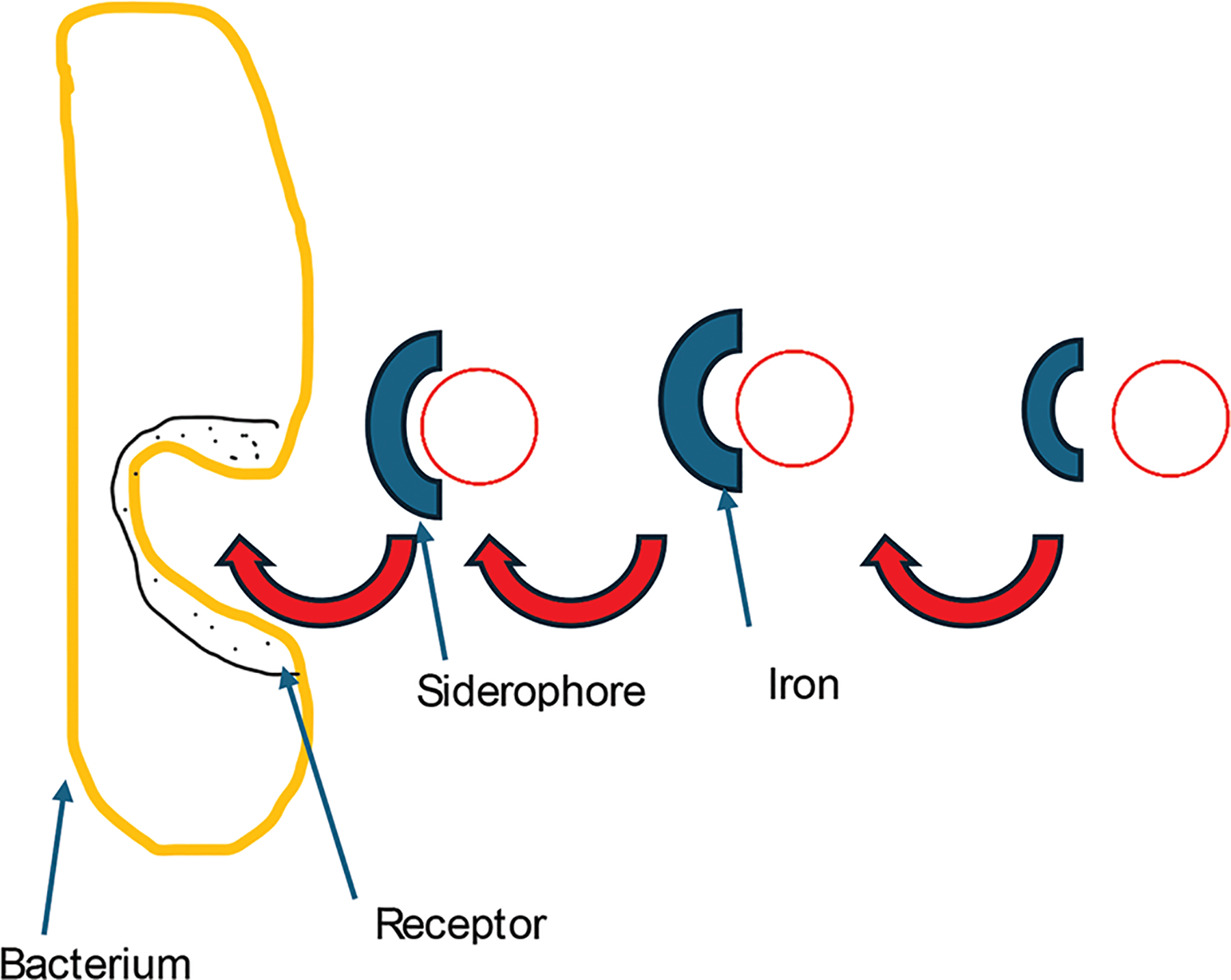
A bacterial cell acquiring iron through siderophores. Bacteria secrete siderophores to scavenge iron from the environment and import it back into the cell through specific outer membrane receptors. Image created by the author.

Microorganisms have adapted to the low bioavailability of iron by secreting siderophores to bind and transport the essential mineral back to the cell (Figure 10).^80^ The types of siderophores used to acquire iron vary among microbes. For example, *E. coli* produces enterobactin (and sometimes aerobactin, yersiniabactin, and salmochelin), *S. aureus* synthesizes staphyloferrin A and B, while *Candida* does not produce its own siderophores but can import and use siderophores produced by other organisms, such as ferrichrome, coprogen, and fusarinine C.^81–83^ To ensure suitable iron levels within the cell and prevent toxicity from excess iron, the production of these siderophores is tightly controlled by the global iron homeostasis regulator, *Fur*.^74^

Excess iron can be damaging under aerobic conditions due to its ability to react with reactive oxygen species, triggering oxidative stress that damages cellular components and ultimately leads to cell death.^83^ This is in addition to a variety of other effects, such as disruption of transcription/translation, damage to the cell wall/membrane, interference with respiration, release of cellular components, and binding to thiol groups.^84^ Even though iron can exert antimicrobial effects, microbes can evolve resistance to iron due to genomic changes, which is a significant aspect of bacterial adaptation, particularly in environments like the human body, where iron availability is often limited or carefully controlled.

In response to iron stress, bacteria exhibit specific genomic adaptations that confer resistance. A primary mechanism involves selective sweeps, which increase the frequency of beneficial mutations in certain genes. For example, during adaptation of *E. coli* to iron(II) stress, selective sweeps have been observed in genes such as *fecA*, *ptsP*, *ilvG*, and others (Tables 6 and 7). These genes encode components of the iron-siderophore transport system, which bacteria use to regulate iron concentration.^85^ Furthermore, when faced with iron toxicity, *E. coli* responds by downregulating the expression of iron uptake genes, such as *cirA and fecA*, to limit the metal’s entry into the cell. This genomic and transcriptional remodeling allows the bacteria to adapt to both iron-deficient and iron-excess conditions.

The similarity between gallium and iron indicates that bacteria with resistance to iron are also tolerant to gallium’s toxicity.^29,85,86^ This is a pleiotropic effect, in which an evolutionary adaptation to resist one substance (excess iron) confers a secondary benefit of resistance to another (gallium). Furthermore, exposure to high iron levels can exert selective pressure, directly driving the evolution of gallium-resistant bacteria.^87^ Mutations in genes like *fecA*, *ptsP*, and *ilvG* can alter a bacterium’s iron transport systems, which are responsible for importing both iron and gallium. The resulting disruption prevents bacteria from importing gallium, thereby conferring resistance. This same change also restricts iron intake, enabling bacteria to better tolerate environments with high iron levels.

**Figure 11.**
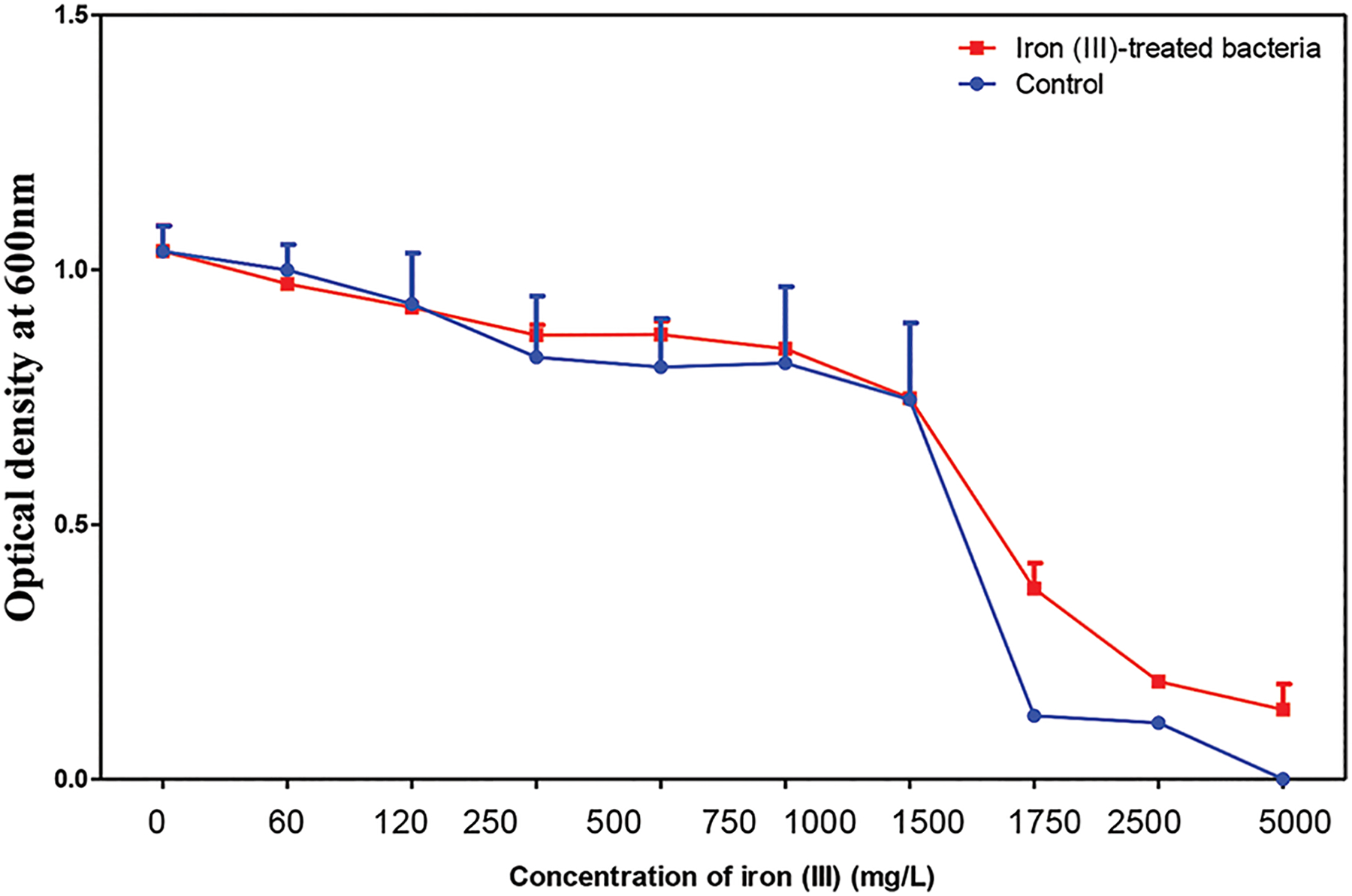
The mean and standard error of the 24-h growth of cultures in increasing concentrations of iron after 30 days. Image created by the author.

**Figure 12.**
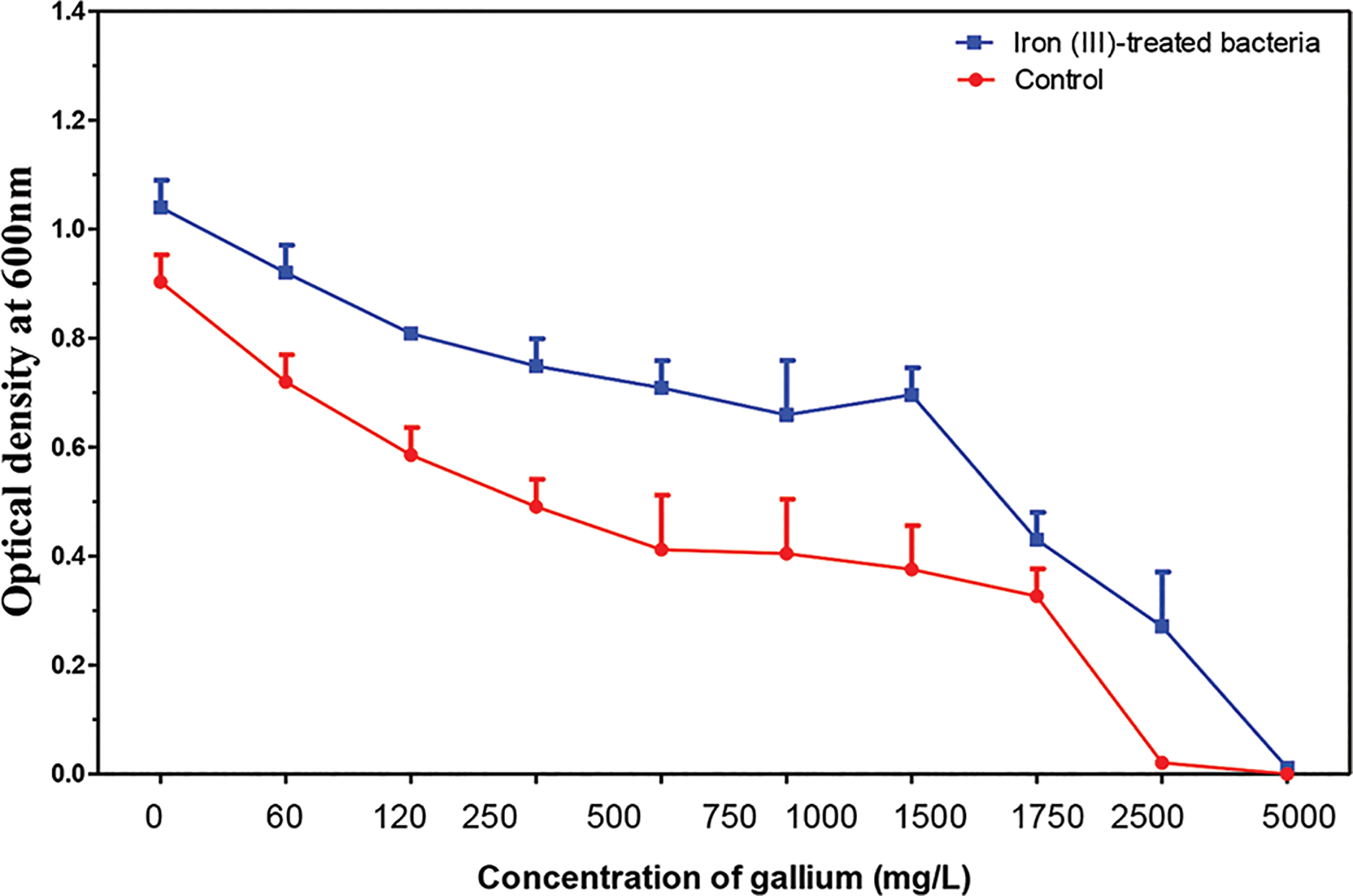
The mean and standard error of 24-h growth for cultures in increasing concentrations of gallium after 30 days. Image created by the author.

As shown in Figure 11, these studies confirm that resistance to iron extends beyond bacterial species. After 30 days of iron exposure, *C. tropicalis* developed gallium resistance, demonstrating a significantly higher 24-h growth rate than the control when cultured in elevated iron concentrations. Iron resistance in *Candida* conferred cross-resistance to gallium’s “Trojan horse” strategy (Figure 12) due to the following reasons: Certain mutations may have inactivated or reduced the efficiency of high-affinity iron uptake systems, thereby limiting both iron and gallium intake. This allows *Candida* to survive gallium exposure by reducing the amount of the toxic metal that enters the cell. Several studies have shown that certain fungi can acquire iron through alternative, siderophore-independent pathways. Two of the most-studied alternative mechanisms are reductive iron assimilation and the direct utilization of the host’s iron.^88–90^ Overexpression of these alternative iron acquisition systems can lead to increased intracellular iron levels, which compete with gallium for binding sites and restore normal iron-dependent metabolic function. Resistance to gallium can be mediated by efflux pumps that transport metal ions out of the cell. Several efflux pumps that manage iron toxicity may also be effective at managing gallium, conferring cross-resistance.^91^ A high-iron environment has been shown to increase *Candida*’s resistance to several conventional antifungal drugs. This indicates a broad link between cellular iron status and drug susceptibility, and mechanisms that help *Candida* tolerate high iron levels could also contribute to gallium resistance.^92–94^

## 5. Conclusion

Leveraging a “Trojan horse” or “Greek gift” strategy, gallium can combat antibiotic-resistant bacteria by mimicking iron and interfering with their metabolism. However, pathogens like *E. coli*, *S*. *aureus*, and *C. tropicalis* can develop gallium resistance. This resistance can be acquired via genetic mutations and morphological changes, as observed in experimental evolution studies. In addition, in cases such as *C. tropicalis*, resistance may arise from temporary, non-heritable phenotypic adjustments rather than permanent genetic changes. Studying how microbes develop gallium resistance is crucial for developing new antimicrobial treatments, addressing the rising threat of antibiotic resistance, and understanding the fundamental biological mechanisms underlying microbial iron management.

## Figures and Tables

**Table 1.** Selective sweeps in 10-day gallium-resistant *Escherichia coli* cultures

Gene	Position	Mutation	Annotation	Description of genes
*qmcA* ←/→ *fetA*	515,859	C to G	Intergenic (−86/−60)	PHB domain membrane-anchored putative protease/iron exporter
*fepD* ←/→ *entS*	622,244	IS5 (−) +4 bp	Intergenic (−55/−53)	Iron-enterobactin transporter subunit/enterobactin exporter
*nhaB* ←	1,234,632	A to G	L29S (TTA to TCA)	Sodium: proton antiporter
*ydfE* →	1,650,461	C to A	Pseudogene (384/765 nt)	Qin prophage; pseudogene; phage or prophage related
*yedN* ←	2,011,665	G to T	Pseudogene (376/678 nt)	Pseudogene, IpaH/YopM family
*yfgF* ←/→ *yfgG*	2,629,042	IS5 (+) +4 bp	Intergenic (−104/−245)	Cyclic-di-GMP PDE
*ypjC* ←/← *ileY*	2,785,563	G to A	Intergenic (−552/+199)	Pseudogene/tRNA-Ile
*rpoS* ←	2,866,695	IS1 (+) +9 bp	Coding (849–857/993 nt)	RNA polymerase, sigma S (sigma 38) factor
*prlF* →	3,277,273	(TTCAACA) 2 to 3	Coding (272/336 nt)	Antitoxin of the SohA (PrlF)–YhaV TA system
*agaI* →/→ *yraH*	3,287,319	C to T	Intergenic (+294/−107)	Galactosamine-6-phosphate isomerase
*dcuB* ←/← *dcuR*	4,349,066	T to A	Intergenic (−322/+249)	C4-dicarboxylate transporter, anaerobic; DcuS co-sensor
*insI1* ←	4,507,739	G to T	D293E (GAC to GAA)	IS30 transposase
*fecE* ←	4,510,928	A to T	L177Q (CTG to CAG)	Iron-dicitrate transporter subunit E
*fecB* ←	4,514,119	C to T	D64N (GAT to AAT)	Iron-dicitrate transporter subunit B
*fecA* ←	4,515,480	C to T	G400S (GGC to AGC)	Ferric citrate outer membrane transporter
*fecA* ←	4,516,171	G to C	N169K (AAC to AAG)	Ferric citrate outer membrane transporter

Notes: →Forward strand, ←Reverse strand.

Abbreviations: GMP PDE: Guanosine monophosphate phosphodiesterase; Ile: Isoleucine; nt: Nucleotide; PHB: Prohibitin homology; TA: Toxin-antitoxin.

**Table 2.** Polymorphisms in the *Escherichia coli* control cultures

Gene	Position	Mutation	Annotation	Description of genes
*crl* →	258,768	G to A	Pseudogene (93/331 nt)	Pseudogene, sigma factor-binding protein, stimulates RNA polymerase holoenzyme 1
*yagK* ←	292,571	G to T	Y126^a^ (TAC to TAA)	CP4–6 prophage; conserved protein
*aspC* ←	985,574	G to A	L46L (CTG to TTG)	Aspartate aminotransferase, PLP-dependent
*putA* ←	1,076,773	C to A	A704S (GCT to TCT)	Fused DNA-binding transcriptional regulator/proline dehydrogenase
*puuR* →	1,362,050	C to A	P47H (CCT to CAT)	Repressor for the divergent *puu* operons, putrescine inducible
*yddG* ←	1,547,149	C to T	T7T (ACG to ACA)	Aromatic amino acid exporter
*ydiJ* ←/→ *ydiK*	1,769,067	A to G	Intergenic (−382/−7)	Putative FAD-linked oxidoreductase/UPF0118 family inner membrane protein
*ydiV* ←/← *nlpC*	1,792,237	A to C	Intergenic (−217/+30)	Anti-FlhD4C2 factor, inactive EAL family phosphodiesterase
*torZ* ←	1,955,394	C to T	A538A (GCG to GCA)	Trimethylamine N-oxide reductase system III, catalytic subunit
*elaD* →	2,383,257	Δ1 bp	Coding (545/1212 nt)	Protease, capable of cleaving an AMC–ubiquitin model substrate
*trmN* ←	2,712,451	A to G	I105T (ATT to ACT)	tRNA1(Val) (adenine (37)-N6)-methyltransferase
*yqeI* →	2,988,528	C to A	I9I (ATC to ATA)	Putative transcriptional regulator
*lysS* ←	3,034,615	C to T	R187H (CGC to CAC)	Lysine tRNA synthetase, constitutive
*frlR* →	3,504,511	T to C	Y154H (TAT to CAT)	Putative DNA-binding transcriptional regulator
*arsC* →/→ *yhiS*	3,651,029	IS2 (+) +5 bp	Intergenic (+367/−258)	Arsenate reductase/pseudogene
*aslB* →	3,983,887	G to A	M310I (ATG to ATA)	Putative AslA-specific sulfatase-maturating enzyme
*cyaA* →	3,991,328	A to T	K59I (AAA to ATA)	Adenylate cyclase
*rhaB* ←	4,097,105	(TGT) 3 to 2	Coding (342–344/1470 nt)	Rhamnulokinase
*sthA* ←	4,160,216	G to A	A192V (GCG to GTG)	Pyridine nucleotide transhydrogenase, soluble
*rpoB* →	4,182,820	C to T	H526Y (CAC to TAC)	RNA polymerase, beta prime subunit
*rpoB* →	4,183,378	T to C	S712P (TCC to CCC)	Protein inhibitor of RNase E/putative acetyltransferase
*rpoB* →	4,183,379	C to T	S712F (TCC to TTC)	RNA polymerase, beta subunit
*rpoC* →	4,186,532	A to G	K395E (AAA to GAA)	RNA polymerase, beta prime subunit
*rraB* →/← *yjgM*	4,479,020	A to T	Intergenic (+131/+14)	Protein inhibitor of RNase E/putative acetyltransferas
*ahr* ←/→ *leuX*	4,496,351	C to T	Intergenic (−142/−54)	Aldehyde reductase, NADPH-dependent, Zn-containing, broad specificity/tRNA-Leu

Notes:

a Variants highlighted for interpretive or functional relevance. →Forward strand, ←Reverse strand.

Abbreviations: AMC: 4-aminomethylcoumarin; FAD: Flavin adenine dinucleotide; Leu: Leucine; NADPH: Nicotinamide adenine dinucleotide phosphate (reduced form); nt: Nucleotide; PLP: Pyridoxal 5’-phosphate; Val: Valine.

**Table 3.** Selective sweeps in 30-day gallium-resistant *Staphylococcus aureus* cultures

Gene	Position	Mutation	Annotation	Description of genes
*sfaA/sfaD*	2,228,365	G to A	Intergenic (−35/−67)	Staphyloferrin A export MFS transporter/D-ornithine--citrate ligase
*KQ76_RS01520*	342,330	T to C	I180I^a^ (ATT to ATC)	DUF3169 family protein
*fmtA*	1,017,325	C to T	Q304^a^ (CAA to TAA)	Teichoic acid D-Alanine esterase
*KQ76_RS08360*	1,700,478	G to A	G59S^a^ (GGC to AGC)	Adenine phosphoribosyltransferase
*dltB*	860,322	G to A	E344K^a^ (GAA to AAA)	Peptidoglycan teichoic acid D-alanyltransferase
*hssR*	2,389,192	G to C	R188P^a^ (CGA to CCA)	DNA-binding heme response regulator
*rsp*	2,412,575	T to G	G84D^a^ (GGT to GAT)	AraC family transcriptional regulator
*KQ76_RS11280/KQ76_RS11285*	2,252,747		Intergenic (−54/+157)	M23 family metallopeptidase/HAD IIB family hydrolase
*KQ76_RS13020*	2,574,726	G to C	G69A^a^ (GGC to GCC)^c^	Alpha/beta hydrolase
*KQ76_RS13825*	2,746,925	C to G	A172G^a^ (GCT to GGT)	ECF-type riboflavin transporter substrate-binding protein
*KQ76_RS12955*	2,564,194	G to C	A17P^a^ (GCA to CCA)	Hypothetical protein
*gltB*	453,752		Pseudogene (4457/4500 nt)	Glutamate synthase large subunit
*KQ76_RS13475*	2,665,478	A to T	E162V^a^ (GAA to GTA)	Glutathione peroxidase
*purS*	1,027,271	G to A	A87P^a^ (GCA to CCA)	Phosphoribosylformylglycinamidine synthase subunit
*KQ76_RS11175*	2,233,551	C to G	T39S^a^ (ACT to AGT)	BCCT family transporter
*KQ76_RS10985*	2,190,680	A to T	T500S^a^ (ACG to TCG)	BglG family transcription antiterminator
*KQ76_RS12180*	2,408,435	T to A	L213I^a^ (TTA to ATA)	Magnesium transporter CorA family protein
*graR*	673,306	G to C	A185P^a^ (GCA to CCA)	Response regulator transcription factor GraR/ApsR
*KQ76_RS07375*	1,536,460	T to A	Y112^a^ (TAT to TAA)	Phage major capsid protein
*KQ76_RS09255*	1,890,405	G to C	S137T^a^ (AGT to ACT)	Hypothetical protein
*KQ76_RS11185*	2,236,063	G to C	R52P^a^ (CGT to CCT)	NADP-dependent oxidoreductase
*KQ76_RS04770*	986,849	A to T	K56^a^ (AAA to TAA)	ATP-binding protein
*ylqF*	1,213,003	G to C	E184D^a^ (GAG to GAC)	Ribosome biogenesis GTPase
*smpB*	810,694	T to A	M1K^a^ (ATG to AAG)^b^	SsrA-binding protein
*vraE*	2,766,496	A to T	I591I^a^ (ATA to ATT)	Peptide-resistance ABC transporter permease subunit
*KQ76_RS02795*	596,886	C to T	A144V^a^ (GCA to GTA)	Uracil-DNA glycosylase
*KQ76_RS01360/lip2*	311,944		Intergenic (−69/−348)	YjiH family protein/YSIRK domain-containing triacylglycerol lipase Lip2/Geh
*capA*	116,233	G to A	V151M^a^ (GTG to ATG)	Capsular polysaccharide-type 5/8 biosynthesis protein
*KQ76_RS04220*	871,433	C to T	T26I^a^ (ACA to ATA)	FAD/NADP-binding protein
*KQ76_RS05175/KQ76_RS05180*	1,066,987		Intergenic (−157/+27)	Nramp family divalent metal transporter/YktB family protein
*KQ76_RS01815*	391,361	G to T	S36I^a^ (AGT to ATT)	General stress protein
*mnmG*	2,776,116	T to A	H117Q^a^ (CAT to CAA)	tRNA uridine-5-carboxymethylaminomethyl (34) synthesis enzyme
*thrS*	1,740,864	T to A	L149^a^ (TTA to TAA)	Threonine-tRNA ligase

Notes:

a Mutations highlighted for functional or interpretive relevance;

b Mutations associated with predicted loss of initiation codon;

c Mutations flagged during variant quality filtering for potential technical artifacts.

Abbreviations: ABC: ATP-binding cassette; ATP: Adenosine triphosphate; BCCT: Betaine/carnitine/choline transporter; DUF: Domain of unknown function family; ECF: Energy-coupling factor; FAD: Flavin adenine dinucleotide; GTPase: Guanosine triphosphatase; HAD IIB: Haloacid dehalogenase superfamily, subfamily IIB; MFS: Major facilitator superfamily; NADP: Nicotinamide adenine dinucleotide phosphate.

**Table 4.** Selective sweeps in 30-day *Staphylococcus aureus* control cultures

Gene	Position	Mutation	Annotation	Description of genes
*pstC*	1,384,254	A to T	S178C^a^ (AGT to TGT)	Phosphate ABC transporter permease subunit
*KQ76_RS04365*	907,688	A to T	N549I^a^ (AAT to ATT)	Acyltransferase family protein
*KQ76_RS10525*	2,102,321	G to A	G175E^a^ (GGA to GAA)	PP2C family protein–serine/threonine phosphatase
*KQ76_RS13020*	2,574,726	G to C	G69A^a^ (GGC to GCC)	Alpha/beta hydrolase
*ylqF*	1,213,003	G to C	E184D^a^ (GAG to GAC)	Ribosome biogenesis GTPase
*hssR*	2,389,188	G to C	E187Q^a^ (GAA to CAA)	DNA-binding heme response regulator
*pxpB/greA*	1,672,825		Intergenic (−76/+250)	5-oxoprolinase subunit PxpB/transcription elongation factor
*icaR*	2,727,937	C to T	Q79^a^ (CAA to TAA)	*ica* operon transcriptional regulator
*cozEb*	1,352,269	T to A	I247N^a^ (ATT to AAT)	Cell elongation protein
*mprF*	1,354,114	G to A	G299D^a^ (GGT to GAT)	Bifunctional lysylphosphatidylglycerol flippase/synthetase
*KQ76_RS13825*	2,746,925	C to G	A172G^a^ (GCT to GGT)	ECF-type riboflavin transporter substrate-binding protein
*KQ76_RS12190/rsp*	2,412,155		Intergenic (+1318/170)	YbgA family protein/AraC family transcriptional regulator
*KQ76_RS10985*	2,190,680	A to T	T500S^a^ (ACG to TCG)	BglG family transcription antiterminator
*KQ76_RS09255*	1,890,405	G to C	S137T^a^ (AGT to ACT)	Hypothetical protein
*KQ76_RS12955*	2,564,194	G to C	A17P^a^ (GCA to CCA)	D-lactate dehydrogenase
*ald/KQ76_RS08720*	1,776,393		Intergenic (−57/−84)	Alanine dehydrogenase/universal stress protein
*KQ76_RS04275/KQ76_RS04280*	881,138		Intergenic (+150/−158)	M23 family metallopeptidase/HAD IIB family hydrolase
*mnmG*	2,776,116	T to A	H117Q^a^ (CAT to CAA)	tRNA uridine-5-carboxymethylaminomethyl (34) synthesis enzyme
*ald/KQ76_RS08720*	1,776,393		Intergenic (−57/−84)	Alanine dehydrogenase/universal stress protein
*KQ76_RS12955*	2,564,193	G to C	M16I^a^ (ATG to ATC)	Hypothetical protein
*KQ76_RS13475*	2,665,478	A to T	E162V^a^ (GAA to GTA)	Glutathione peroxidase
*mutS*	1,275,400	G to C	K479N^a^ (AAG to AAC)	DNA mismatch repair protein
*rsp*	2,412,863	G to A	C180Y^a^ (TGT to TAT)	AraC family transcriptional regulator
*smpB*	810,694	T to A	M1K^a^ (ATG to AAG)	SsrA-binding protein
*KQ76_RS01360/lip2*	311,944		Intergenic (−69/−348)	YjiH family protein/YSIRK domain-containing triacylglycerol lipase Lip2/Geh
*KQ76_RS10985*	2,190,680	A to T	T500S^a^ (ACG to TCG)	BglG family transcription antiterminator
*KQ76_RS04770*	986,858	C to A	Q59K^a^ (CAA to AAA)	ATP-binding protein
*KQ76_RS13825*	2,746,925	C to G	A172G^a^ (GCT to GGT)	ECF-type riboflavin transporter substrate-binding protein
*gltB*	453,752		Pseudogene (4457/4500 nt)	Glutamate synthase large subunit
*KQ76_RS12905*	2,554,550	G to C	V173L^a^ (GTC to CTC)	ATP-binding cassette domain-containing protein
*KQ76_RS11175*	2,233,551	C to G	T39S^a^ (ACT to AGT)	BCCT family transporter

Note:

a Variants highlighted for interpretive or functional relevance.

Abbreviations: ABC: ATP-binding cassette; ATP: Adenosine triphosphate; BCCT: Betaine/carnitine/choline transporter; ECF: Energy-coupling factor; GTPase: Guanosine triphosphatase; HAD IIB: Haloacid dehalogenase superfamily, subfamily IIB; PP2C: Protein phosphatase 2C.

**Table 5.** Selective sweeps in 30-day gallium-treated *Candida tropicalis* cultures

Gene	Position	Mutation	Annotation	Description of genes
*sfaA/sfaD*	2,228,365	A to T	Intergenic (−35/−67)	Staphyloferrin A export MFS transporter/D-ornithine–citrate ligase
*KQ76_RS08360*	1,700,478	G to A	G59S (GGC to AGC)	Adenine phosphoribosyltransferase
*fmtA*	1,017,325	C to T	Q304^a^ (CAA to TAA)	Teichoic acid D-Ala esterase
*KQ76_RS01520*	342,330	T to C	I180I (ATT to ATC)	DUF3169 family protein
*KQ76_RS13020*	2,574,726	G to C	G69A (GGC to GCC)^c^	Alpha/beta hydrolase
*hssR*	2,389,192	G to C	R188P (CGA to CCA)	DNA-binding heme response regulator
*ylqF*	1,213,003	G to C	E184D (GAG to GAC)	Ribosome biogenesis GTPase
*dltB*	860,322	G to A	E344K (GAA to AAA)	Peptidoglycan teichoic acid D-alanyltransferase
*KQ76_RS11280/KQ76_RS11285*	2,252,747		intergenic (54/+157)	M23 family metallopeptidase/HAD-IIB family hydrolase
*KQ76_RS01815*	391,361	G to T	S36I (AGT to ATT)	General stress protein
*smpB*	810,694	T to A	M1K (ATG to AAG)^b^	Ssr-Abinding protein
*KQ76_RS07375*	1,536,460	T to A	Y112^a^ (TAT to TAA)	Phage major capsid protein
*purS*	1,027,271	G to C	A87P (GCA to CCA)	Phosphoribosylformylglycinamidine synthase subunit
*mnmG*	2,776,116	T to A	H117Q (CAT to CAA)	RNA uridine-5-carboxymethylaminomethyl (34) synthesis enzyme

Notes:

a Mutations highlighted for functional or interpretive relevance;

b Mutations associated with predicted loss of initiation codon;

c Mutations flagged during variant quality filtering for potential technical artifacts.

Abbreviations: DUF: Domain of unknown function family; GTPase: Guanosine triphosphatase; HAD IIB: Haloacid dehalogenase superfamily, subfamily IIB; MFS: Major facilitator superfamily.

**Table 6.** Adaptation to iron (II) stress drives significant genomic changes at day 200 (*Escherichia coli*)

Gene	Position	Mutation	Annotation	Description
*murC* →	100,804	C to T	P14S (CCC to TCC)	UDP N acetylmuramate: L alanine ligase
*cueR* →	514,008	G to C	V6L (GTA to CTA)	Copper-responsive regulon transcriptional regulator
*yeaG* →	1,868,229	C to T	A441V (GCA to GTA)	Protein kinase, endogenous substrate unidentified; autokinase
*fliP* →	2,021,984	C to A	S39^a^ (TCG to TAG)	Flagellar biosynthesis protein
*ptsP* ←	2,966,878	G to T	C519^a^ (TGC to TGA)	Fused PTS enzyme: PEP protein phosphotransferase (enzyme I)/GAF domain-containing protein
*ilvL* →/→ *ilvX*	3,950,466	G to T	Intergenic (+46/41)	ilvG operon leader peptide/uncharacterized protein
*ilvG* →	3,951,606	+C	Pseudogene (65/663 nt)	Pseudogene, acetolactate synthase 2 large subunit
*fecA ←*	4,515,003	C to T	A559T (GCT to ACT)	Ferric citrate outer membrane transporter
*fecA ←*	4,515,951	C to A	G243C (GGC to TGC)	Ferric citrate outer membrane transporter

Notes:

a Variants highlighted for interpretive or functional relevance. →Forward strand, ←Reverse strand.

Abbreviations: GAF: cGMP-specific phosphodiesterases, adenylyl cyclases, and FhlA domain; nt: Nucleotide; PEP: Phosphoenolpyruvate; PTS: Phosphotransferase system; UDP: Uridine diphosphate.

**Table 7.** Adaptation to iron (III) stress drives significant genomic changes at day 200 (*Escherichia coli*)

Gene	Position	Mutation	Annotation	Description
*dnaK* →	12,614	G to T	R151L (CGT to CTT)	Chaperone Hsp70, with co-chaperone DnaJ
*dnaK* →	13,460	A to C	Q433P (CAG to CCG)	Chaperone Hsp70, with co-chaperone DnaJ
*dnaK* →	13,874		Coding (1712/1917 nt)	Chaperone Hsp70, with co-chaperone DnaJ
*ompF* ←/← *asnS*	987,104		Intergenic (122/+481)	Outer membrane porin 1a (Ia; b; F)/asparaginyl tRNA synthetase
*ompC* ←	2,311,899		A284D (GCT to GAT)	Outer membrane porin 1a (Ia; b; F)/asparaginyl tRNA synthetase
*nudF* ←*/*→ *tolC*	3,178,051		Intergenic (141/61)	ADP-ribose pyrophosphatase/transport channel
*tolC* →	3,178,288		Coding (174/1482 nt)	Transport channel
*tdcR →/→ yhaB*	3,267,652		Intergenic (+54/202)	L-threonine dehydratase operon activator protein/uncharacterized protein
*nlpl* ←/← *pnp*	3,308,463		Intergenic (−39/+68)	Purine nucleoside phosphorylase
*nusA ←*	3,316,755	C to T	R258C (CGT to TGC)	Transcription termination/antitermination L factor
*nusA ←*	3,316,954	T to GC	R191R (CGT to CGC)	Transcription termination/antitermination L factor
*crp* →	3,486,122	G to A^a^	M1M (ATG to ATA)^a^	cAMP-activated global transcription factor, mediator of catabolite repression
*crp* →	3,486,175	G to A	C19Y (TGC to TAC)	cAMP-activated global transcription factor, mediator of catabolite repression
*yjiH* ←	4,560,352	A to G	Q110R (CAG to CGG)	Nucleoside recognition pore and gate family putative inner membrane transporter

Notes:

a Variants flagged during quality control as potentially ambiguous or requiring further validation. →Forward strand, ←Reverse strand.

Abbreviations: ADP: Adenosine diphosphate; nt: Nucleotide.

## Data Availability

Data are available on request from the corresponding author.
